# Hydrogen inhalation and intrathecal magnesium sulfate ameliorate ischemia by suppressing cortical spreading depolarization in a rat subarachnoid hemorrhage model

**DOI:** 10.1016/j.neurot.2025.e00617

**Published:** 2025-05-28

**Authors:** Toru Yoshiura, Satoko Kawauchi, Sho Nishida, Sho Sato, Daichi Hagita, Arumu Endo, Masaya Nakagawa, Takashi Fujii, Yohei Otsuka, Yumiko Mishima, Kazuya Fujii, Satoru Takeuchi, Arata Tomiyama, Terushige Toyooka, Shunichi Sato, Kojiro Wada

**Affiliations:** a Department of Neurosurgery, National Defense Medical College, 3-2 Namiki, Tokorozawa, Saitama 359-8513, Japan; b Division of Bioinformation and Therapeutic Systems, National Defense Medical College Research Institute, 3-2 Namiki, Tokorozawa, Saitama 359-8513, Japan; c Department of Neurosurgery, Social Medical Corporation Shijinkai Ken-o-Tokorozawa Hospital, 4-2692-1 Higashisayamagaoka, Tokorozawa, Saitama 359-1106, Japan

**Keywords:** Early brain injury, Cortical spreading ischemia, Delayed cerebral ischemia, Reactive oxygen species, Free radical scavenger, NMDA receptor

## Abstract

This study investigated whether inhaled hydrogen and intrathecal magnesium could mitigate cortical spreading depolarization and delayed cerebral ischemia in a rat model of subarachnoid hemorrhage. Adult male rats underwent subarachnoid hemorrhage induction with nitric oxide synthase inhibition and high-potassium application to elicit cortical spreading depolarization. Animals were assigned to sham, control, H_2_, Mg, or combined H_2_ and Mg treatment groups. We measured direct current potentials, cerebral blood flow, brain water content, bodyweight changes, and neurological outcomes. Compared with controls, the H_2_ and Mg groups had significantly reduced total depolarization and hypoperfusion times. The combined treatment produced similar benefits. H_2_ alone rapidly shortened depolarization duration, suggesting that it may offer neuroprotection until Mg effects fully manifest. Neither treatment altered physiological parameters, brain water content, bodyweight, or neurological deficits. These findings indicate that H_2_ and Mg reduce key pathophysiological processes related to early brain injury and delayed cerebral ischemia following subarachnoid hemorrhage, potentially improving outcomes by minimizing depolarization events and associated ischemia. H_2_ therapy may provide early protective effects before Mg exertion.

## Graphical abstract

**Figure:**
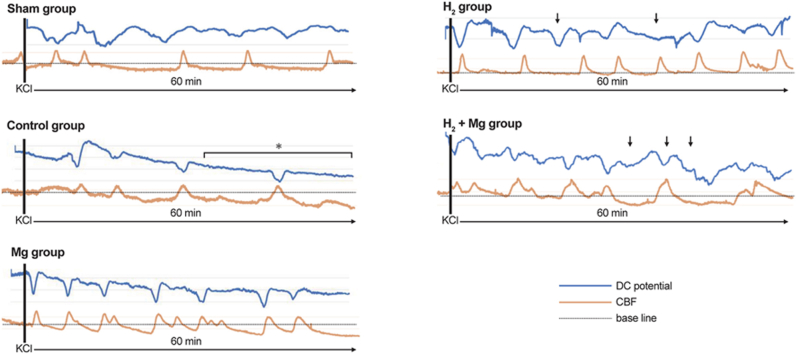

## Introduction

Subarachnoid hemorrhage (SAH), predominantly resulting from the rupture of cerebral aneurysms, is associated with a poor prognosis and a mortality rate ranging from 30 ​% to 40 ​%. Following SAH, early brain injury (EBI) is initiated, which can subsequently lead to delayed cerebral ischemia (DCI), contributing to severe complications or death [1]. Key factors in the pathophysiology of EBI include catecholamine surge, intracranial pressure hyperactivity, intracranial hypoperfusion, microcirculatory failure, inflammatory responses, and cortical spreading depolarization (CSD). CSD, a wave of neuronal and glial depolarization, is strongly linked to the development of EBI and DCI [2,3]. Approximately 80 ​% of patients exhibit CSD within 15 days following SAH [4]. Moreover, if the total duration of depolarization exceeds 180 ​min within 24 ​h of onset, the likelihood of delayed infarction increases to approximately 80 ​% [4].

CSD is a propagating depolarization event within the central nervous system [[5], [6], [7], [8], [9]]. In the context of SAH, the leakage of hemoglobin and potassium from erythrocytes initiates rapid ion influx and efflux into neuronal and glial cells, leading to a near-total loss of membrane potential [[5], [6], [7], [8], [9]]. Consequently, repolarization, or the recovery of membrane potential, demands significant energy [[5], [6], [7], [8], [9]]. Additionally, CSD in SAH has been shown to promote cortical spreading ischemia (CSI), further contributing to brain injury [7,10,11]. This process is intricately linked to the neurovascular response during CSD, commonly referred to as neurovascular coupling (NVC) [[12], [13], [14], [15]]. Upon the onset of CSD, the cerebral vascular response varies between healthy and pathological brain tissue. In healthy tissue, blood flow typically increases, whereas in pathological conditions such as SAH, there is often a marked reduction in blood flow [6,11,[16], [17], [18]]. This diminished blood flow is thought to exacerbate ongoing cerebral ischemia and prolong the duration of CSD [[19], [20], [21], [22]]. A prolonged CSD duration may subsequently elevate the production of reactive oxygen species (ROS) within mitochondria, thereby exacerbating tissue damage [23,24].

Hydrogen is renowned for its potent antioxidative effects and rapid diffusion through tissues, making it highly safe and effective as a free radical scavenger in biological systems [25]. In contrast, magnesium, which acts as an N-methyl-d-aspartate (NMDA) receptor antagonist and possesses vasodilator properties, has been reported to effectively suppress CSD and CSI when administered intrathecally in a rat SAH model [26]. Additionally, it has been shown that intracisternal administration of a magnesium solution, combined with intravenous hydrogen solution, reduced DCI and improved functional outcomes in humans following severe SAH. However, it takes 2–3 days for magnesium to reach effective concentrations in the cerebrospinal fluid [27]. This suggests that, although magnesium reaches effective concentrations, hydrogen remains effective for DCI and functional outcomes because it is a small molecule that diffuses rapidly. It has been reported to reach therapeutic concentrations during the EBI period (within minutes to 72 ​h after onset) [25] and may suppress EBI by reducing ROS following CSD. However, no studies have yet quantified the effects of hydrogen on CSD and cerebral blood flow (CBF). The purpose of this study was to examine the effects of hydrogen inhalation on CSD and CSI in a rat SAH-mimicking model, as well as its additive effects when combined with intrathecal magnesium administration.

## Methods

### Study design

All experimental procedures in this study were approved by the institutional animal care committees (Approval No. 21057). Adult male Sprague–Dawley rats, aged 8–12 weeks and weighing 300–400 ​g (Japan SLC, Shizuoka, Japan), were housed in a vivarium with a 12-h light/dark cycle and provided unlimited access to food and water. Their rectal temperature was maintained at 37.5 ​± ​0.5 ​°C using a heating pad. The rat SAH-mimicking model was established using the method previously described by Dreier et al. [28]. L-NG-Nitroarginine methyl ester (l-NAME) and a high-potassium solution were used to simulate the post-SAH brain environment, characterized by reduced nitric oxide (NO) and elevated potassium concentrations in the cisternal space, both of which are known to induce CSD. The artificial cerebrospinal fluid (ACSF) used in the study was ARTCEREB (Otsuka Pharmaceutical, Naruto, Tokushima, Japan), composed of Na^+^ 145 ​mM, K^+^ 2.8 ​mM, Mg^2+^ 1.1 ​mM, Ca^2+^ 1.15 ​mM, Cl^−^ 129 ​mM, HCO_3_^−^ 23.1 ​mM, P 1.1 ​mM, and glucose 3.39 ​mM. The l-NAME ​+ ​[K^+^]_ACSF_ solution contained ACSF, 1 ​mM l-NAME, and 35 ​mM ​K^+^ (KCl). The solution for the l-NAME ​+ ​[K^+^]_ACSF_ ​+ ​[Mg^2+^] group included l-NAME ​+ ​[K^+^]_ACSF_ with an additional 5 ​mM ​Mg^2+^ (MgSO_4_). 1.3 ​% H_2_ gas (hydrogen mixed with 21 ​% oxygen and balance nitrogen) was purchased from Taiyo Nippon Sanso JFP Corporation (Kanagawa, Japan) [29]. The 1.3 ​% H_2_ gas was administered via ventilator control. This treatment was initiated on day 0 (the day of SAH onset) for the hydrogen administration groups (H_2_ and H_2_ ​+ ​Mg groups), as described below.

**Fig. 1:**
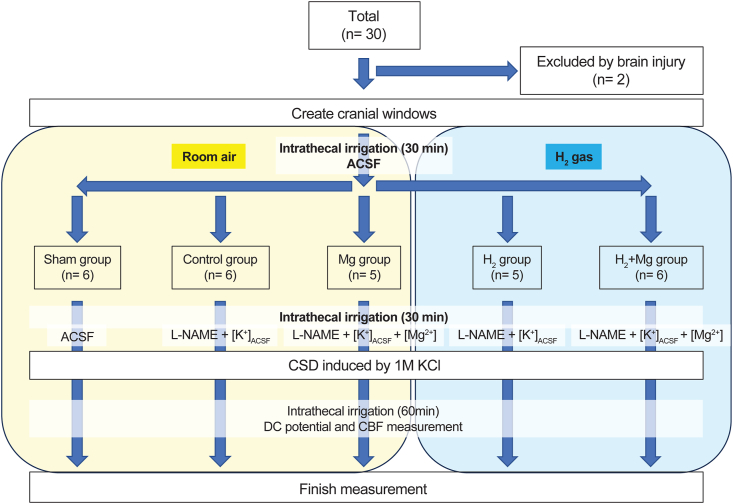
Study design. Rats were divided into five groups (sham, control, Mg, H_2_, and H_2_ + Mg groups).

The initial experimental group consisted of 30 rats; however, two were excluded because of accidental brain injury by procedures, leaving a total of 28. These were divided into five groups: sham surgery (sham group; six rats), l-NAME ​+ ​[K^+^]_ACSF_ (control group; six rats), l-NAME ​+ ​[K^+^]_ACSF_ ​+ ​[Mg^2+^] (Mg group; five rats), l-NAME ​+ ​[K^+^]_ACSF_ ​+ ​[H_2_] (H_2_ group; five rats), and l-NAME ​+ ​[K^+^]_ACSF_ ​+ ​[H_2_] ​+ ​[Mg^2+^] (H_2_ ​+ ​Mg group; six rats) (Fig. 1).

### Anesthesia and surgical procedures

Rats were anesthetized with 3 ​% isoflurane in a 79 ​% nitrogen and 21 ​% oxygen gas mixture. Following anesthesia, buprenorphine (0.01 ​mg/kg) was administered intraperitoneally for pain management. Arterial blood pressure was continuously monitored, and blood samples were collected via cannulation of the middle segment of the tail artery. Arterial blood gases, including pH, PaCO_2_, and PaO_2_, were measured after surgery and subsequent CSD induction, as described below. After administering vecuronium bromide (0.15 ​mg/kg), tracheal intubation was performed. The rats were placed on mechanical ventilation, with anesthesia maintained using 1.5–2% isoflurane. For the surgical procedure, the rat's head was secured in a stereotactic frame, and a 1.5-cm midline incision was made to expose the bregma and lambda reference points.

**Fig. 2:**
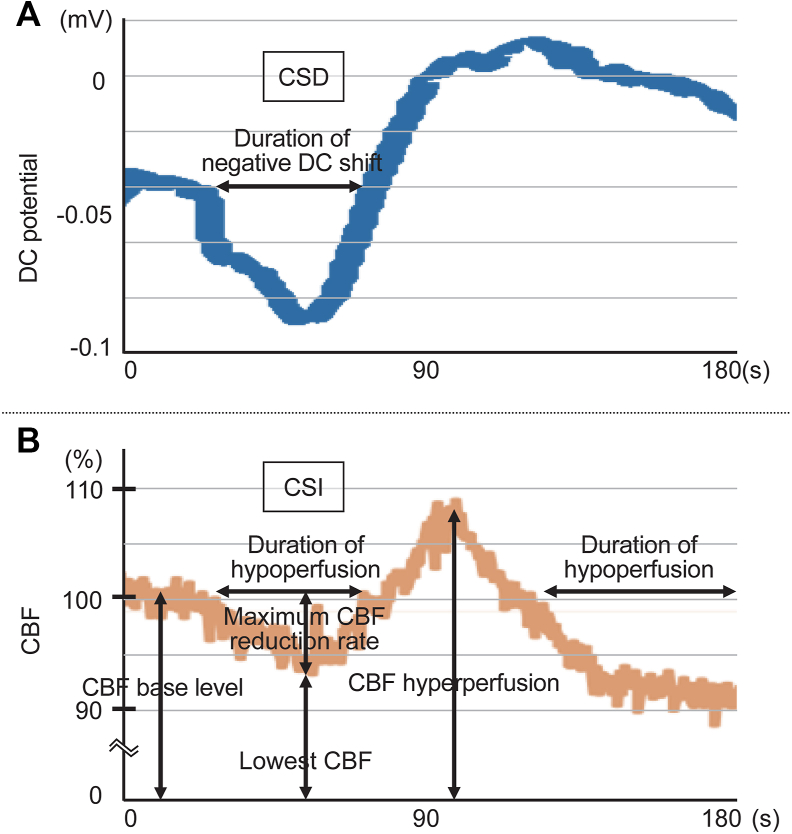
Measurement of DC potential (A) and CBF (B). DC, direct current; CBF, cerebral blood flow; CSI, cortical spreading ischemia; CSD, cortical spreading depolarization.

As reported [26], surgical access to the brain was achieved through a cranial window and two burr holes drilled over the parietal cortex. Specifically, an oval burr hole measuring 3 ​× ​2 ​mm was created 1 ​mm posterior to the bregma, whereas a circular burr hole with a diameter of 2 ​mm was placed 3 ​mm lateral to the lambda. Additionally, a 3-mm cranial window was created 1 ​mm anterior and 3 ​mm lateral to the lambda. The oval burr hole was used to measure direct current (DC) potential, the circular burr hole served as the site for placing a KCl-soaked cotton ball to induce CSD, and the cranial window was used for irrigation and measuring CBF. Following removal of the dura mater under the circular burr hole and cranial window, the latter was sealed with a cover glass to create a closed cranial window. An Ag–AgCl electrode with a 1.5-mm tip diameter was placed on the cortical surface via the oval burr hole to record extracellular DC potential. The cortical surface beneath the closed window was irrigated continuously with ACSF for 30 ​min. This was followed by three distinct irrigation protocols: ACSF alone (sham group), l-NAME ​+ ​[K^+^]_ACSF_ (control and H_2_ groups), and l-NAME ​+ ​[K+]ACSF ​+ ​[Mg2+] (Mg group and H_2_ ​+ ​Mg group), with a 30-min equilibration period for each. CBF was measured by laser speckle flowmetry (OZ- 2; Omegawave, Tokyo, Japan) at the center of the closed cranial window, designated as the region of interest. After baseline measurements of DC potential and CBF were taken for 10 ​min, CSD was induced by applying a 2-mm-diameter cotton ball soaked in 1 ​M KCl solution to the pial surface, replenished with 5 ​μL of the same solution every 20 ​min for 1 ​h. DC potential was continuously recorded throughout this period, and the frequency of CSD events was counted. The duration of each negative DC shift was aggregated to calculate the total depolarization time over the hour. Simultaneously, CBF was continuously monitored, noting episodic reductions indicative of CSI (Fig. 2). The maximum CBF reduction rate was calculated as the percentage drop from baseline to the lowest value recorded during the first CSI episode. The total time of hypoperfusion, representing the cumulative duration of all hypoperfusion events, including CSI episodes within the hour, was also recorded. Long-lasting CSI, which has been strongly correlated with subsequent brain tissue damage in experimental rodent models and clinical studies of SAH [10,28], was defined as CSI episodes lasting more than 20 ​min in this study. These events were specifically monitored. After the experiment, the pial surface was cleaned with ACSF, and DC potential, CBF, and blood pressure were monitored. Following the surgical procedure, the incision sites were sutured, and the rats were kept on ventilation until they fully recovered from anesthesia. Once extubated, the rats were returned to their housing with free access to food and water.

**Fig. 3:**
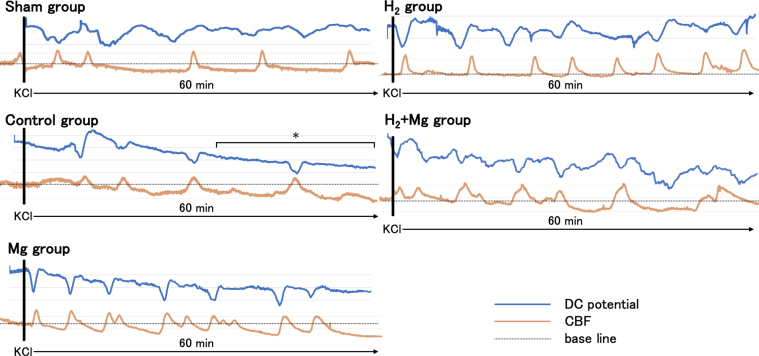
Effect of magnesium (Mg) and hydrogen (H_2_) treatment on direct current (DC) potential and cerebral blood flow (CBF). The blue line is DC potential, the orange line is CBF, and the black dotted line is baseline CBF. Cortical spreading depolarization (CSD) occurrence is indicated by a black arrow. CSD-induced cortical spreading hyperemia in the sham group and cortical spreading ischemia (CSI) in the other groups. Long-lasting CSI (∗) was observed in half of the control group but not in the other groups.

### Data recordings

KCl-induced events were measured using DC potential and electrocorticography signals, which were amplified with a DC amplifier (DAM50; World Precision Instruments, Sarasota, FL) and recorded through an A/D converter (Model ML825; AD Instruments, Bella Vista, Australia). Data, including body temperature, arterial blood pressure, and electrocorticography, were continuously recorded via a data acquisition system for offline analysis (PowerLab; AD Instruments). CBF was recorded through the cranial window using laser speckle flowmetry, as previously described. Image acquisitions and analyses were performed using the provided software (LSI-V314 and LIA-V315; Omegawave).

### Brain water content

Brain edema was assessed by measuring brain water content on the second day following CSD induction. Under deep anesthesia induced by inhalation of 5 ​% isoflurane, the brains were quickly removed. Coronal sections, 4-mm thick, were prepared at a level 1 ​mm anterior and 3 ​mm lateral to the lambda, corresponding to the region where CBF was measured. Only the cerebral cortex of the left hemisphere was sampled. The tissue was immediately weighed (wet weight). After dehydration at 105 ​°C for 48 ​h, the sample was weighed again (dry weight). The brain water content was then calculated using the following formula:

Brain water content (%) ​= ​[(wet weight - dry weight) / wet weight] ​× ​100

### Bodyweight and neurological deficits

Bodyweight was recorded on day 0 and day 2, and the percentage of bodyweight loss was calculated using the formula: bodyweight loss (%) ​= ​[(bodyweight on day 0 - body weight on day 2)/bodyweight on day 0] ​× ​100. Neurological function was assessed on day 2 by an independent observer using the modified Garcia scoring system [30]. The evaluation included the following parameters: spontaneous activity (0–3 points), climbing (1–3 points), body proprioception (1–3 points), and response to vibrissae touch (1–3 points). Animals were assigned a score between 3 and 18, with higher scores indicating better function. Neurological deficits were calculated using the following formula: neurological deficits ​= ​18(full score) – neurological score on day 2.

### Quantification and statistical analysis

All statistical analyses were performed using EZR (Saitama Medical Center, Jichi Medical University, Saitama, Japan), a graphical user interface for R (The R Foundation for Statistical Computing, Vienna, Austria). Specifically, it is a modified version of R Commander designed to incorporate statistical functions frequently used in biostatistics. Data are presented as the mean ​± ​standard error of the mean. All values were analyzed using a one-way analysis of variance followed by the Tukey–Kramer multiple comparison procedure. Statistical significance was determined at P ​< ​0.05.

## Results

### Physiological parameters

No significant differences were observed in baseline body weight, mean arterial blood pressure, or blood gases (pH, PaCO_2_, PaO_2_) across the groups at any point during the surgical procedures (Table 1).

**Table 1:** Physiological parameters.

Variable	Sham Group (n = 6)	Control Group (n = 6)	H_2_ Group (n = 5)	Mg Group (n = 5)	H_2_ + Mg Group (n = 6)	P Value
Bodyweight	354.17 ± 36.53	328.33 ± 39.71	347.00 ± 39.94	343.00 ± 29.92	378.33 ± 33.27	NS
Arterial blood gas						
pH						
Intubation	7.39 ± 0.06	7.40 ± 0.04	7.38 ± 0.03	7.39 ± 0.03	7.40 ± 0.03	NS
Pre-CSD	7.34 ± 0.07	7.40 ± 0.04	7.35 ± 0.02	7.47 ± 0.02	7.39 ± 0.02	NS
Post-CSD	7.38 ± 0.00	7.39 ± 0.06	7.34 ± 0.04	7.45 ± 0.02	7.39 ± 0.03	NS
PaCO_2_(mmHg)						
Intubation	45.7 ± 6.4	40.5 ± 7.8	42.3 ± 3.9	40.8 ± 5.1	38.5 ± 4.4	NS
Pre-CSD	53.2 ± 14.9	35.5 ± 3.5	41.2 ± 3.8	31.7 ± 3.6	40.9 ± 3.1	NS
Post-CSD	43.3 ± 1.4	34.9 ± 5.2	42.0 ± 6.1	32.1 ± 4.3	39.7 ± 3.8	NS
PaO_2_(mmHg)						
Intubation	74.0 ± 17.3	79.0 ± 10.4	82.5 ± 0.7	69.5 ± 12.8	92.7 ± 12.5	NS
Pre-CSD	75.0 ± 29.7	80.0 ± 3.5	80.0 ± 2.8	80.0 ± 4.7	104.8 ± 51.6	NS
Post-CSD	93.0 ± 5.7	75.3 ± 4.5	73.0 ± 1.4	88.0 ± 7.8	99.0 ± 31.2	NS
MABP (mmHg)						
Baseline	98.1 ± 8.5	84.1 ± 5.1	91.3 ± 5.0	88.7 ± 11.6	94.1 ± 9.7	NS
20 min	86.8 ± 38.8	87.2 ± 5.8	90.3 ± 4.3	96.1 ± 10.9	89.9 ± 13.0	NS
40 min	100.8 ± 14.0	87.5 ± 7.6	91.8 ± 5.6	96.4 ± 16.6	93.9 ± 14.6	NS
60 min	102.0 ± 11.9	88.8 ± 7.8	91.7 ± 6.8	95.4 ± 19.4	97.2 ± 14.4	NS

Values are expressed as mean ± standard error of the mean. Physiological parameters did not differ significantly between the groups (one-way analysis of variance followed by the Tukey-Kramer multiple comparison procedure). CSD, cortical spreading depolarization; PaCO_2_, arterial carbon dioxide tension; PaO_2_, arterial oxygen tension; MABP, mean arterial blood pressure; NS, not significant.

### Change in DC potential and change in CBF

Topical application of KCl induced a negative DC shift and changes in CBF in all groups. CSD-induced cortical spreading hyperemia in the sham and H_2_ groups, and CSI in the control and Mg groups. Long-lasting CSI was observed in half of the control group but not in any other groups (Table 2).

**Table 2:** Incidence of long-lasting cortical spreading ischemia.

Sham Group	Control Group	H_2_ Group	Mg Group	H_2_ + Mg Group
0/6	3/6	0/5	0/5	0/6

### Incidence of CSD events

**Fig. 4:**
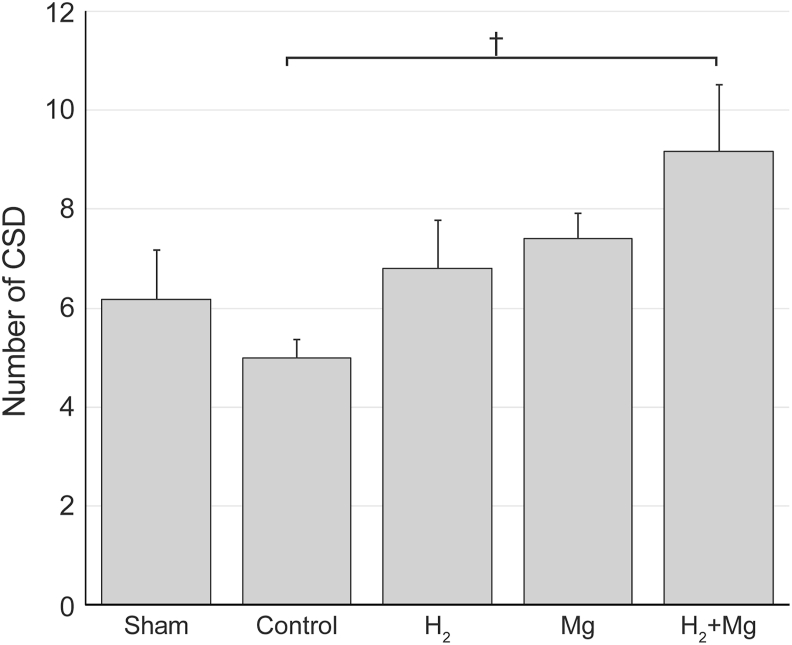
The number of CSD events was significantly higher in the H_2_ + Mg group than in the control group (P < 0.05). CSD, cortical spreading depolarization.

The average number of CSD events during the 1-h period was 6.2 ​± ​1.01 in the sham group, 5.0 ​± ​0.37 in the control group, 6.8 ​± ​0.97 in the H_2_ group, 7.4 ​± ​0.51 in the Mg group, and 9.2 ​± ​1.35 in the H_2_ ​+ ​Mg group (Fig. 4). The number of CSD events was significantly higher in the H_2_ ​+ ​Mg group than in the control group (P ​< ​0.05). Although the number of CSD events in the H_2_ and Mg groups was higher than in the control group, the differences did not reach statistical significance.

### Incidence of CSI events

**Fig. 5:**
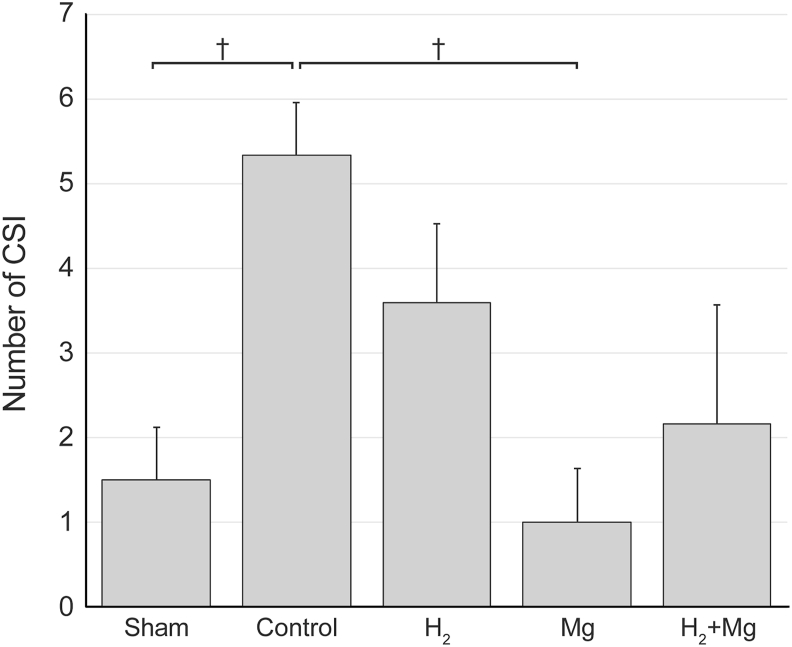
The numbers of CSI events in the Mg group and sham group were significantly smaller than that in the control group (P < 0.05). CSI, cortical spreading ischemia.

The average number of CSI events during the 1-h period was 1.5 ​± ​0.62 in the sham group, 5.3 ​± ​0.61 in the control group, 3.6 ​± ​0.93 in the H_2_ group, 1.0 ​± ​0.63 in the Mg group, and 2.2 ​± ​1.40 in the H_2_ ​+ ​Mg group (Fig. 5). The numbers of CSI events in the Mg and sham groups were significantly smaller than that in the control group. The numbers of CSI events in the H_2_ and H_2_ + Mg groups were smaller than that in the control group but did not reach statistical significance.

### Total depolarization time

**Fig. 6:**
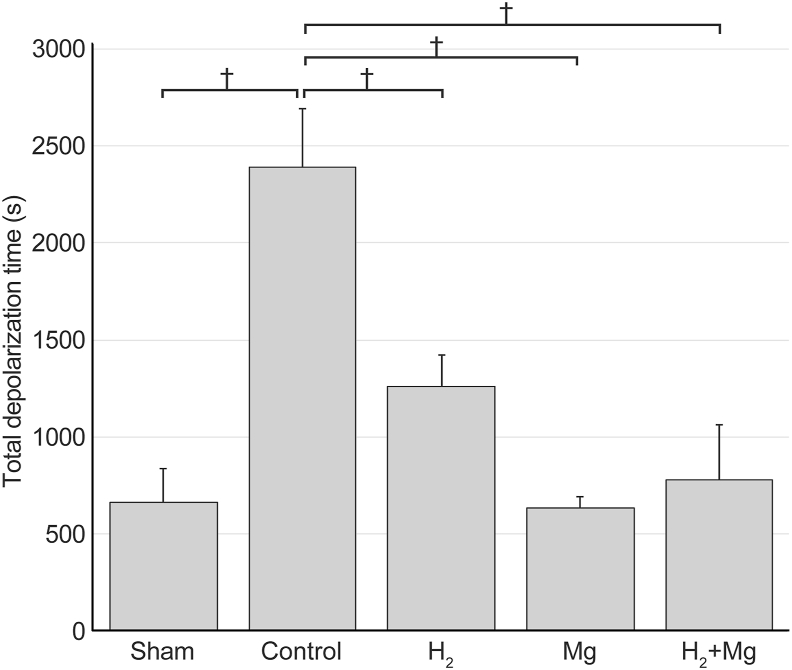
The total depolarization times in the H_2_, Mg, and H_2_ + Mg groups were significantly shorter than that in the control group (P < 0.05).

The total depolarization time was 666 ​± ​170 ​s in the sham group, 2389 ​± ​305 ​s in the control group, 1262 ​± ​162 ​s in the H_2_ group, 633 ​± ​57 ​s in the Mg group, and 778 ​± ​s 283 in the H_2_ ​+ ​Mg group (Fig. 6). The total depolarization time in the control group was significantly longer than that in the sham group (P ​< ​0.05), whereas those in the H_2_, Mg, and H_2_ ​+ ​Mg groups were significantly shorter than that in the control group (P ​< ​0.05). No significant differences were found between the sham, H_2_, Mg, and H_2_ + Mg groups.

### Total hypoperfusion time

**Fig. 7:**
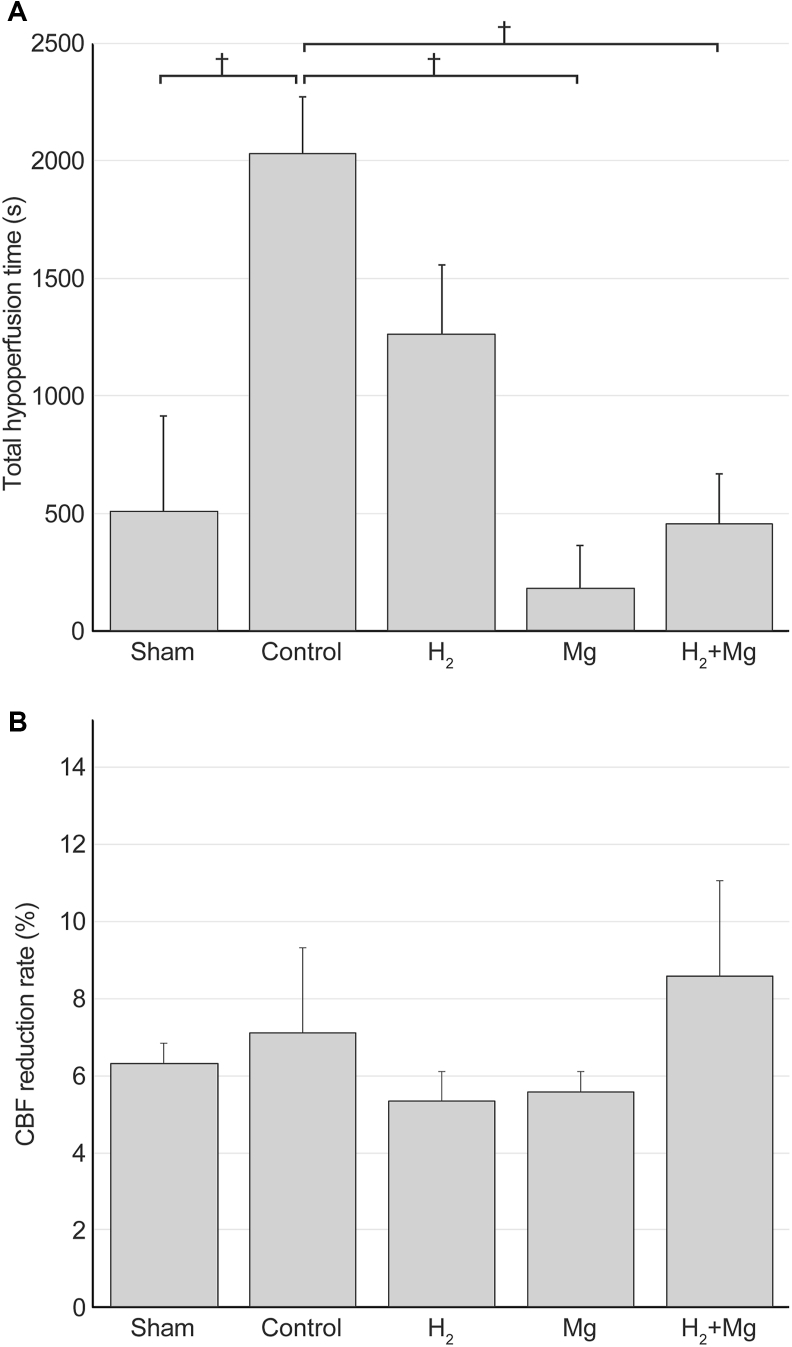
(A) The total hypoperfusion times in the Mg and H_2_ + Mg groups were significantly shorter than that in the control group (P < 0.05). (B) The maximum cerebral blood flow (CBF) reduction rate was not significant in any group.

The total hypoperfusion time was 510 ​± ​405 ​s in the sham group, 2033 ​± ​239 ​s in the control group, 1263 ​± ​293 ​s in the H_2_ group, 182 ​± ​182 ​s in the Mg group, and 456 ​± ​211 ​s in the H_2_ ​+ ​Mg group (Fig. 7A). The total hypoperfusion times in the Mg, H_2_ ​+ ​Mg, and sham groups were significantly shorter than that in the control group (P ​< ​0.05). Long-lasting CSI was observed in half of the control group but not in the H_2_, Mg, or H_2_ ​+ ​Mg groups, as mentioned previously. The maximum CBF reduction rate was not significantly different across any of the groups (Fig. 7B).

### Brain water content

**Fig. 8:**
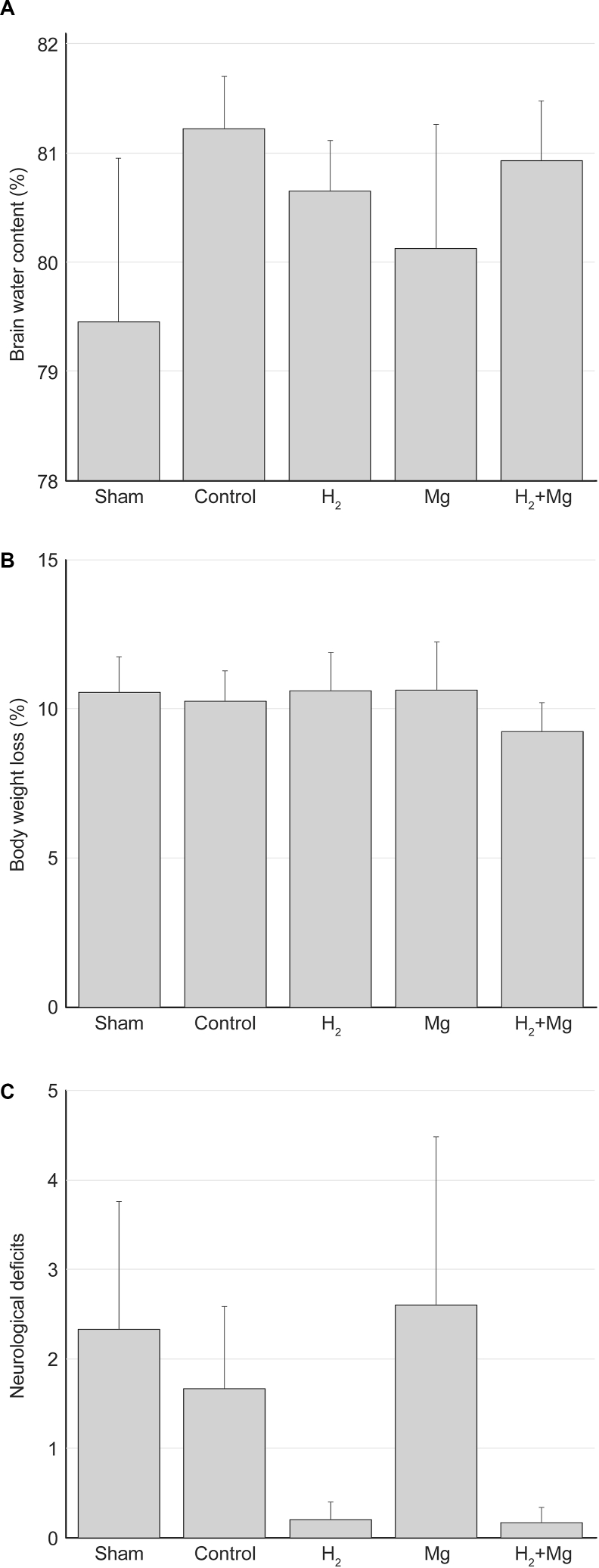
(A) There were no significant differences in brain water content across groups. (B) Bodyweight loss was not significant in any group. (C) Neurological deficits were not significant in any group.

Brain water content in the sham group was the lowest, whereas the control group exhibited the highest brain water content (Fig. 8A). However, no significant differences were found across the groups (P ​= ​0.675).

### Bodyweight loss and neurological deficits

The H_2_ ​+ ​Mg group showed the least bodyweight loss, although no significant differences were observed between the groups (Fig. 8B, P ​= ​0.906). Neurological deficits were lower in the H_2_ and H_2_ ​+ ​Mg groups than in the other groups. However, no significant differences were found across the groups (Fig. 8C, P ​= ​0.402).

## Discussion

In this study, the total depolarization time was significantly reduced in the Mg, H_2_, and H_2_ ​+ ​Mg groups than in the control group (Fig. 6). Furthermore, no long-lasting CSI was observed in the CBF of the Mg, H_2_, and H_2_ ​+ ​Mg groups (Fig. 3). These findings suggest that magnesium and hydrogen alone effectively suppressed long-lasting CSI by reducing sustained depolarization. It has been demonstrated that CSD in SAH induces spreading ischemia as a result of prolonged depolarization and elevated extracellular potassium ion concentrations, leading to an energy metabolism imbalance. This imbalance results from decreased oxidative substrates, such as glucose, and increased energy demand. Consequently, the Na–K pump in neurons is inhibited, which triggers a vicious cycle of calcium influx into mitochondria, increased ROS production, and decreased ATP synthesis, thereby sustaining neuronal depolarization and exacerbating spreading ischemia [6,23,24]. In this study, hydrogen reduced the total depolarization time, potentially altering the blood flow response to normal NVC and preventing long-lasting CSI, which could otherwise contribute to EBI and DCI. The main effect of hydrogen gas is an antioxidant effect on ROS and reactive nitrogen species (RNS). Pleiotropic effects, such as regulation of microglial activity and suppression of inflammatory cytokines, have also been reported. These are thought to act directly or indirectly to form pharmacological effects [31], and many reports of EBI reduction by hydrogen have been published [[32], [33], [34], [35]]. However, there have been no reports on the effect of hydrogen on total depolarization time. To the authors’ best knowledge, this study is the first to report the effect of hydrogen on total depolarization time and CSI, providing new insights into EBI mitigation.

Interestingly, as an additive to intrathecal magnesium administration, the number of CSD events was significantly increased in the H_2_ ​+ ​Mg group than in the control group (Fig. 4). This finding suggests that, in addition to the effects of hydrogen described above, the vasoconstrictive inhibitory effect of magnesium may have contributed to stopping the vicious cycle following CSD. In other words, by suppressing sustained depolarization and improving energy metabolism, their combined effects may have facilitated a transition of the tissue from a refractory state—where the cortex is less responsive to subsequent stimuli as a result of metabolic and ionic disturbances during or after CSD—to a state in which the tissue is again responsive, enabling another depolarization event to occur. In this study, the number of CSI events was significantly lower in the Mg group than in the control group (Fig. 5), and the total hypoperfusion time was significantly shorter in the Mg and H_2_ ​+ ​Mg groups than in the control group (Fig. 7A). The occurrence of long-lasting CSI was also suppressed, suggesting that magnesium improves CBF, as reported previously [26]. Regarding magnesium, its effects as an NMDA-type glutamate receptor antagonist and vasodilator have demonstrated potential for reducing infarct size and improving CSD in animal ischemic models [[36], [37], [38]]. Magnesium functions as a vasodilator in cerebral and small arteries [39] and functions as a calcium-channel blocker with antiplatelet properties. Furthermore, it has been reported to protect neurons by inhibiting glutamate release and blocking NMDA receptors [[39], [40], [41], [42]]. Previous studies have indicated that intrathecal administration of magnesium solution reduces DCI and improves functional outcomes in patients following severe aneurysmal SAH [27,43]. However, it should be considered that intrathecal administration may take time to achieve effective therapeutic concentrations, as discussed later.

In this study, there were no significant differences in brain water content, a measure of cerebral edema and a key pathology of EBI among the groups. However, brain edema tended to be milder in the H_2_- and Mg-treated groups than in the control group. The brain water content values for all groups were consistent with those reported in previous studies [28,33,44]. The anti-edema effect observed may be attributed to the combined reducing effect of hydrogen and the NMDA receptor inhibition effect of magnesium. In this model, a SAH-mimicking environment was induced in a localized region of the cortex via irrigation procedures, which may explain the lack of statistically significant differences in brain water content across the groups.

The SAH-mimicking rat model used in this study simulates the brain environment after SAH by inducing a decrease in NO and an increase in potassium concentration in the cerebral cistern through the administration of l-NAME and a high-potassium solution. Alternative models of SAH, such as autologous blood infusion into the cistern or perforation of cerebral arteries, are also commonly used. However, our model is advantageous as it reproduces a localized SAH environment confined to the cerebral cortex, offering greater stability with less variation in injury severity between subjects. Nevertheless, it is important to note that this model does not replicate SAH in the entire brain cistern. As a result, the severity of the injury is milder compared to that in full SAH models, which may explain the absence of significant differences in body weight loss and neurological function.

The CSI waveforms do not represent the typical pattern previously reported using the same model [26]. This discrepancy may result from subtle differences in the ionic composition of the ACSF and [K^+^]_ACSF_ solutions.

The DC potential is measured at a site different from the perfusion site, meaning that KCl-induced CSDs and CSDs originating from the perfusion site may be recorded [45]. However, since CBF is measured directly at the perfusion site, and long-lasting CSI suppression has been observed following H_2_ and Mg administration, these findings suggest that H_2_ and Mg improve NVC at the perfusion site by suppressing sustained depolarization, thus restoring cortical excitability and making the tissue more susceptible to subsequent CSD events.

Based on the findings of this study, hydrogen therapy emerges as a promising next-generation therapeutic strategy for SAH. Although the antioxidant capacity of hydrogen gas is lower than that of vitamin C, a well-known antioxidant, hydrogen selectively neutralizes highly reactive and cytotoxic radicals, such as hydroxyl radicals and peroxynitrite, which are among the most damaging ROS/RNS. Notably, hydrogen gas does not interact with ROS/RNS that serve physiological roles, such as hydrogen peroxide and NO [46]. Additionally, hydrogen gas is easy to administer and cost-effective, making it a feasible treatment for patients in the acute phase following SAH. Because of its high tissue diffusibility, hydrogen is effective not only through inhalation but also via alternative routes, such as oral consumption of hydrogen-rich water or intravenous administration, further supporting its clinical applicability [46]. Clinical trials evaluating hydrogen gas inhalation in patients with SAH are currently underway [47]. Although magnesium therapy has been shown to suppress DCI, it typically takes 2–3 days for magnesium to reach effective concentrations in the cerebrospinal fluid [27]. In contrast, hydrogen reaches therapeutic concentrations almost immediately [25] and may provide early suppression of EBI by inhibiting sustained depolarization and reducing total depolarization time, potentially bridging the gap until magnesium therapy becomes effective in human patients.

## Conclusions

In a rat SAH-mimicking model, the combined use of hydrogen gas inhalation and intrathecal magnesium administration significantly reduced total depolarization and total hypoperfusion times. Hydrogen gas alone also significantly reduced total depolarization time and did not induce long-lasting CSI. These findings suggest that hydrogen gas inhalation may reduce total depolarization time and mitigate EBI in patients with SAH until the therapeutic effects of magnesium treatment are fully established.

## Data availability statement

The data that support the findings of this study are available from the corresponding author upon reasonable request.

## Author contributions

Toru Yoshiura: Writing-Original Draft, Methodology, Investigation, Formal Analysis, Conceptualization.

Satoko Kawauchi: Methodology, Investigation, Conceptualization.

Sho Nishida: Methodology, Investigation.

Sho Sato: Writing- Reviewing and Editing.

Daichi Hagita: Writing- Reviewing and Editing.

Arumu Endo: Writing- Reviewing and Editing, Investigation, Formal Analysis.

Masaya Nakagawa: Writing- Reviewing and Editing, Investigation, Formal Analysis.

Takashi Fujii: Writing- Reviewing and Editing.

Yohei Otsuka: Writing- Reviewing and Editing, Investigation, Formal Analysis.

Yumiko Mishima: Writing- Reviewing and Editing.

Kazuya Fujii: Writing- Reviewing and Editing.

Satoru Takeuchi: Writing- Reviewing and Editing.

Arata Tomiyama: Writing- Reviewing and Editing.

Terushige Toyooka: Writing- Reviewing and Editing.

Shunichi Sato: Writing- Reviewing and Editing, Investigation, Supervision, Conceptualization.

Kojiro Wada: Writing- Reviewing and Editing, Investigation, Supervision, Conceptualization.

## Funding

This research did not receive any specific grant from funding agencies in the public, commercial, or not-for-profit sectors.

## Declaration of competing interest

The authors declare that they have no known competing financial interests or personal relationships that could have appeared to influence the work reported in this paper.
